# Assessment of Deep Partial Thickness Burn Treatment with Keratin Biomaterial Hydrogels in a Swine Model

**DOI:** 10.1155/2016/1803912

**Published:** 2016-11-29

**Authors:** D. Poranki, C. Goodwin, M. Van Dyke

**Affiliations:** ^1^Wake Forest Institute for Regenerative Medicine, Wake Forest School of Medicine, Medical Center Boulevard, Winston-Salem, NC 27157, USA; ^2^Department of Biomedical Engineering and Mechanics, Virginia Polytechnic Institute and State University, Blacksburg, VA 24061, USA

## Abstract

Partial thickness burns can advance to full thickness after initial injury due to inadequate tissue perfusion and increased production of inflammatory cytokines, which has been referred to as burn wound progression. In previous work, we demonstrated that a keratin biomaterial hydrogel appeared to reduce burn wound progression. In the present study, we tested the hypothesis that a modified keratin hydrogel could reduce burn wound progression and speed healing. Standardized burn wounds were created in Yorkshire swine and treated within 30 minutes with keratin hydrogel (modified and unmodified), collagen hydrogel, or silver sulfadiazine (SSD). Digital images of each wound were taken for area measurements immediately prior to cleaning and dressing changes. Wound tissue was collected and assessed histologically at several time points. Wound area showed a significant difference between hydrogels and SSD groups, and rates of reepithelialization at early time points showed an increase when keratin treatment was used compared to both collagen and SSD. A linear regression model predicted a time to wound closure of approximately 25 days for keratin hydrogel while SSD treatment required 35 days. There appeared to be no measurable differences between the modified and unmodified formulations of keratin hydrogels.

## 1. Introduction

Burns are one of the most catastrophic injuries to treat. Often, partial thickness burns convert to full thickness due to inadequate tissue perfusion and increased production of inflammatory cytokines leading to protein denaturation and necrosis [1]. The phenomenon of burn injury progression occurs when biochemical events downstream of the original damage cause further tissue loss. As a result, wounds increase in surface area and deepen, progressing from second-degree to third-degree burns and increasing overall total body surface area (TBSA) burned, thereby increasing risk to the patient, healing time, and treatment expense. In 1953, Jackson described a model of burn injury in terms of three zones of tissue damage: (1) the inner zone of coagulation, which is characterized by necrotic tissue, (2) an intermediate zone of stasis, which is characterized by damaged cells that may live or die depending on treatment and other factors, and (3) an outer zone of hyperemia where cells are stressed but will likely survive if the wound does not get infected and standard treatment is given [2].

Superficial and partial thickness burns heal from the remaining epithelial structures in the dermis [3]; therefore, the zone of stasis represents an opportunity for treatment as these cells are capable of survival if an appropriate therapy can be developed that restores normal cell function. To date, limited clinical research has been directed in this area. In vivo studies, however, have shown that treatments like cerium nitrate, curcumin, activated protein C (APC), recombinant tissue type plasminogen (r-TPA), TAK-044 (nonselective endothelin receptor antagonist), simvastatin, and beraprost sodium (prostaglandin I2 analogue) were able to control burn injury progression in rats by preventing tissue necrosis in the zone of stasis [4–11]. In general, these studies have made use of drug compounds that require intravenous or enteric administration, thus complicating their clinical development. The use of topical dressings made from biomaterials and agents in wound care has largely been directed toward chronic wounds [12, 13]. Topical application of an efficacious therapeutic agent targeting cell survival in the zone of stasis in burns represents a simplified, local treatment modality, but little work has been published toward this goal.

Previous studies in our lab have shown that a biomaterial hydrogel made from a heterogeneous mixture of extracted keratin proteins (termed “crude keratose”) increased tissue sparing and enhanced wound healing in a pilot study using both mice and swine [14]. As part of the study, an in vitro thermal injury model using primary mouse dermal fibroblasts showed that the so-called “gamma keratose” fraction from this heterogeneous mixture of keratin proteins was able to maintain increased cell viability compared to the other treatments post thermal stress. Mass spectrometry analysis identified the major constituents of the gamma keratose fraction as keratins 81, 83, 85, 86, 31, 33A, 33B, and 34 with lower than expected molecular weights, suggesting that these were degraded alpha keratin proteins [15]. As such, they represent distinct peptides, potentially with biological function different than that of their parent molecules. A second study showed that gamma keratose was able to salvage the cells by downregulating many genes involved in cell death pathways [16]. In the present study, we hypothesized that the peptides found in the gamma keratose fraction would provide a therapeutic benefit when used in a topical keratin biomaterial hydrogel formulation. This postulate was tested by comparing a crude keratose hydrogel (Crude KOS) with a reformulated hydrogel, modified keratose hydrogel (MKH), which contained a reduced amount of gamma keratose, in a swine burn injury model. The amount of gamma keratose was reduced to reflect the concentration at which gamma keratose was effective in the heat stress cell culture model [16]. A commercial collagen hydrogel and the clinical standard of care, silver sulfadiazine cream (SSD), were used as controls.

## 2. Methods

### 2.1. Keratin Protein Extraction

Keratin protein was extracted from human hair fibers using the oxidative protocol specified by de Guzman et al. [17] The extracted keratin is referred to as crude keratose extract.

### 2.2. Separation of Alpha and Gamma Keratose Fractions

Crude keratose extract was further separated into alpha and gamma keratose fractions by isoelectric precipitation. Concentrated hydrochloric acid was added dropwise to the crude keratose extract solution with stirring until a pH of 4.2 was reached. At this pH, the alpha keratose precipitates leaving the acid-soluble gamma fraction in solution. The insoluble alpha keratose fraction was separated by fixed angle centrifugation at 10,000 rpm for 40 min at 4°C. After neutralizing the gamma-containing supernatant to pH 8.4, it was dialyzed against endotoxin free water using a 5 kilodalton (kDa) nominal low molecular weight cutoff spiral wound cartridge (Millipore, Billerica, MA) for 5 volume washes. Finally, the solution was concentrated to minimal volume, pH adjusted to 7.4, lyophilized, and sterilized by gamma irradiation at 1 MRad prior to use. The precipitated alpha keratose was redissolved in 0.1 M sodium hydroxide (NaOH) solution and dialyzed against endotoxin free water using a 5 kDa spiral wound cartridge (Millipore) for 5 volume washes. Finally, the solution was concentrated to minimal volume, pH adjusted to 7.4, lyophilized, and sterilized by gamma irradiation at 1 MRad prior to use.

### 2.3. Hydrogel Preparation

The MKH was prepared using a proprietary mixture of alpha and gamma keratose fractions and the lyophilized powder reconstituted with phosphate buffered saline (PBS) to form a hydrogel. Crude keratose extract lyophilized powder was similarly reconstituted with PBS to form a 15% w/v hydrogel of Crude KOS. The gels were made under sterile conditions, centrifuged to remove air bubbles, and incubated in a warm room (37°C) overnight on a shaker. Gels were loaded into syringes for easy application onto the burn wounds. A collagen hydrogel (Woun'Dress® Collagen Hydrogel, Coloplast) and SSD was obtained from commercial sources and used as received.

### 2.4. Swine Burn Study

The study was approved by the Wake Forest University Animal Care and Use Committee (ACUC). Fourteen female Yorkshire swine (Baux-Mountain, Winston-Salem, NC) weighing 20–25 kg at the time of the burn procedure were used in this study. The animals were housed in individual pens upon their arrival and allowed to become acclimated for at least 7 days.

### 2.5. General Anesthesia and Monitoring

The animals were washed and shaved one day prior to the burn procedure, at which time they were pretreated with a transdermal fentanyl patch (50 mcg/h) for pain management. While on study, the animals were fasted each night prior to the administration of anesthesia. Anesthesia was induced intramuscularly with ketamine (10 mg/kg) and dexmedetomidine (0.05 mg/kg). Isoflurane was used to maintain the animal in an anesthetic state during the burn procedure and dressing changes. The previously applied fentanyl patch was removed and a new patch (75 mcg/h) was applied during the burn procedure and dressing changes, which occurred every 3 days. Blood pressure, heart rate, and body temperature were monitored during anesthesia for any complications.

### 2.6. Burn Wound Creation

Deep partial thickness burns were created using a pressure controlled burn device as previously described [18]. This spring loaded device was made of insulative Delrin® containing cylindrical brass blocks (360 grade) measuring 3 cm in diameter and 5 cm in height that were used to create the burn wounds. The blocks were heated in a boiling solution of polyethylene glycol (PEG 400; Sigma-Aldrich, St. Louis, MO) and deionized (DI) water (4 : 1 ratio). A stainless steel post protruded from the top of each block to allow for easy insertion into the burn device holder. A total of three identical brass blocks were rotated for use during the burn procedure to assure temperature equilibration and minimize the amount of time needed to maintain the animal under general anesthesia. Twelve burns were created on each swine, six on either side of the mid dorsal line between the shoulder and hip using a brass block contact time of 20 seconds. Two swine were assigned to each terminal time point, with each of the 24 wounds randomly assigned to and divided evenly among the four treatment groups. Treatments included Crude KOS, MKH, Woun'Dress (collagen hydrogel; herein referred to as “Coloplast”), and SSD, which were administered within 30 minutes after creating the burns. Each treatment had six replicate wounds at each terminal time point for histological assessments (i.e., *n* = 6). Following the treatments, the wounds were covered with saline soaked, nonadhesive gauze (Telfa, Tyco Healthcare, Mansfield, MA), followed by an occlusive adhesive dressing (Ioban™ 2, 3M, St. Paul, MN), a fabric stocking, a protective plastic shield, and a nylon jacket to keep the dressings in place and prevent the animal from rubbing directly on the wounds. Atipamezole (0.05 mg/kg) was administered intramuscularly to reverse the effect of the dexmedetomidine after burning. Dressing changes were performed every three days under general anesthesia, where wound cleaning involved carefully “dabbing” excess exudate within and surrounding the wound with saline-soaked gauze, as well as debriding loose eschar by hand or with forceps. Attached wound eschar and reepithelialized tissue was left intact. Two animals were euthanized at 1, 3, 6, 9, 12, 15, and 30 days after the burn procedure and tissues were harvested for histological analysis.

### 2.7. Digital Images of Wounds

Digital images were captured on the day of burning and 1, 3, 6, 9, 12, 15, 21, and 30 days postburn during the dressing changes, immediately after cleaning and debriding the wounds (Nikon-D90, Nikon Inc., Melville, NY). All of the images were taken with the camera placed at the same distance and the same angle (90° perpendicular) relative to the wound. A metric ruler and grey scale were positioned in the plane of the wound during image capture for later processing and calibration of the images as described by other investigators [19]. Two-dimensional wound area was measured from these surface images using ImageJ software (National Institutes of Health, Bethesda, MD). These data were represented as mean ± standard deviation (SD).

### 2.8. Histology

The entire wound with a border of normal skin was excised from the animal at each designated time point and one-half of the wound was fixed in 10% neutral buffered formalin for 2 days. After fixation, each excised wound (including both burned and adjacent normal tissue) was cut exactly into quarters for ease of processing. A tissue cross section was cut from an inner edge of one of the quarter pieces and positioned closest to the center of the wound. Using this method, each tissue section presented data from the outer border of the wound to its center. The tissue was then processed in 70% isopropyl alcohol (IPA), 80% IPA, 95% IPA, 100% IPA, and xylene (Leica ASP300S Tissue processor, Buffalo Grove, IL) overnight. The tissue was embedded in paraffin, cut in 5 *μ*m sections using a microtome (Leica CM 1850, Leica Microsystems Inc., Buffalo Grove, IL), and stained with hematoxylin and eosin (H&E). Representative sections of each wound were evaluated histomorphometrically by a blinded reviewer.

#### 2.8.1. Burn Depth Measurement

Digital images of stained tissue sections were captured (Axio Imager M1 Microscope, Carl Zeiss USA, Thornwood, NY). The thickness of the normal skin section from the epidermal surface down to the dermal-fat junction and the residual (uninjured) dermal section beneath the burned region were measured. An average of three thickness measurements were taken within each section, evenly spaced to account for variation in animal anatomy. Burn depth was determined by calculating the difference in thickness of the residual dermis beneath the burned region from the thickness of the normal skin adjacent to the burn region (typically ca. 6 mm in this study).

#### 2.8.2. Granulation Tissue Measurement

The thickness of new granulation tissue (characterized by connective tissue and blood vessels stained pink with H&E) was determined by measuring from the epidermal surface to the depth of granulation tissue present. An average of three depth measurements obtained along the length of the stained tissue sections as previously described. The depth of granulation tissue measured relative to the thickness of normal skin (epidermal and dermal) tissue was used to determine % granulation tissue.

#### 2.8.3. Reepithelialization Measurement

The burned region without epithelial cell coverage (epithelial coverage being defined as at least one layer of cells staining dark pink) was measured from the stained tissue sections. The amount of reepithelialized tissue present was determined by subtracting the burned region from the measured radius of the wound (as determined from the digital image for the same wound at that time point; measured radii were approximately 1.5 cm in length). A ratio of the length of reepithelialized tissue relative to the measured radius of the wound determined % reepithelialization. Reepithelialization data was analyzed in two phases: early time points that included days 3 and 6 and later time points that included days 12, 15, and 30. This was done because the reepithelialization curves demonstrated two-phase healing behavior, suggesting that wound healing rates were different between these two time ranges and that distinct phases of healing (tissue salvage and cell survival followed by cell proliferation, matrix deposition, and tissue remodeling) were present.

### 2.9. Data Collection and Statistical Analysis

For visual wound assessment of digital images, wound size values for each treatment group were averaged (e.g., *n* = 36 on day 3, *n* = 30 on day 6, and *n* = 24 on day 9) and reported as mean ± SD. For burn depth and granulation tissue assessment, values for each treatment group from six replicates were averaged and presented as mean ± SD. A two-way analysis of variance (ANOVA) with a Bonferroni post hoc analysis was performed using Prism (GraphPad Inc., La Jolla, CA) in order to assess significance (*P* > 0.05) in time and/or treatment. Percent reepithelialization was divided into early (day 0 to 6) and late (day 12 to 30) phases. Data were analyzed using a linear regression model and differences in slope (i.e., reepithelialization rate) assessed for significant differences using Prism. For clarity, wound closure was defined as 100% reepithelialization (confirmed by histologic assessment of 100% coverage of at least one layer of confluent epithelial cells), which may have occurred before the 30-day time point in some cases. Time to wound closure was calculated by extrapolating a linear model that included all time points to 100% reepithelialization.

## 3. Results

### 3.1. Visual Wound Assessment

**Figure 1:**
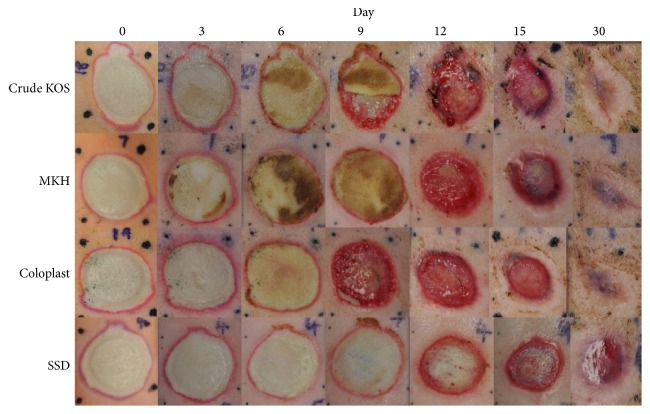
Digital images of healing burns. Images of wounds immediately after burn and at days 3, 6, 12, 15, and 30 correlate with morphometric results. All biomaterial treatments appear to facilitate complete wound closure by 30 days while SSD shows healthy granulation but only a thin layer of new epithelium.

**Figure 2:**
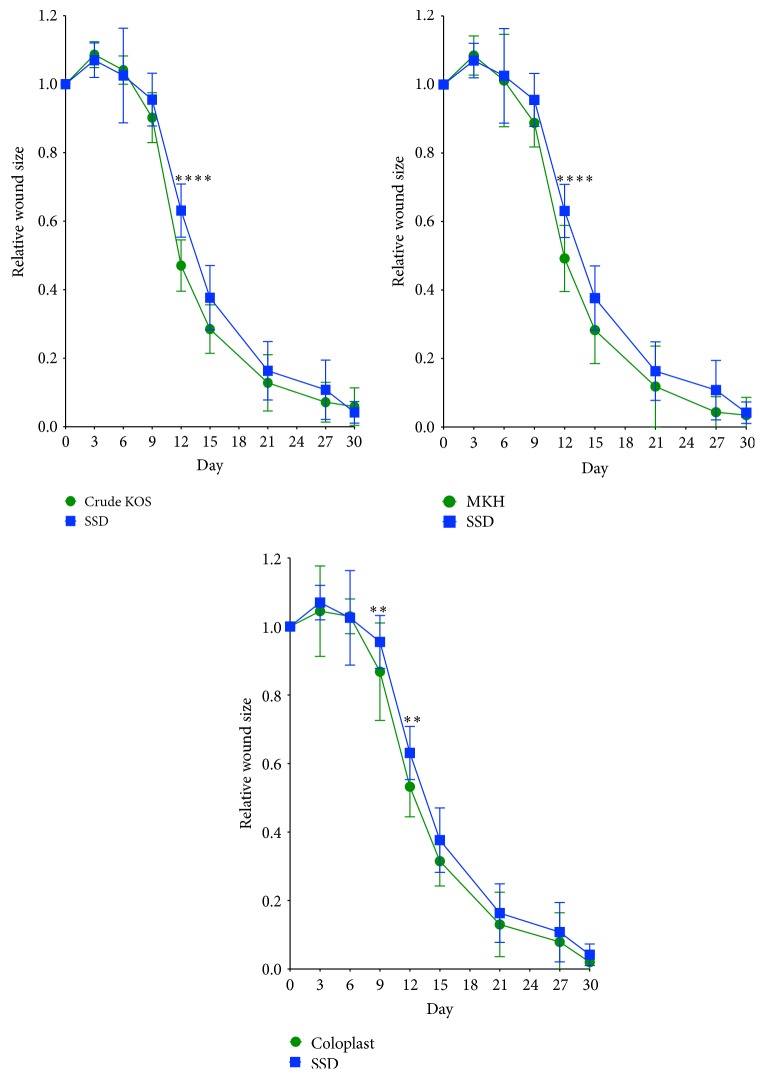
Visual wound assessment. Crude KOS, MKH, and Coloplast all appeared to promote wound healing faster than SSD with highly significant differences at 12 days (Crude KOS and MKH) and significant differences at 9 and 12 days (Coloplast) compared to SSD (^*∗∗*^ *P* < 0.01; ^*∗∗∗∗*^ *P* < 0.001; *n* = 36/across 12 pigs on day 3; *n* = 30/across 10 pigs on day 6; *n* = 24/across 8 pigs on day 9; *n* = 18/across 6 pigs on day 12; *n* = 12/across 4 pigs on day 15; *n* = 6/across 2 pigs on day 30).

The eschar (dead skin) typically sloughs off completely within 9–12 days, so reepithelialization was observed most distinctly in the digital images of the wounds from 9 to 30 days (Figure 1). Digital image analysis data showed significant differences for both Crude KOS and MKH compared to SSD on day 12 (*P* < 0.001) and between Coloplast and SSD on days 9 and 12 (*P* < 0.01) (Figure 2). Most wounds in the hydrogel groups (i.e., Crude KOS, MKH, and Coloplast) were completely closed by 30 days while most SSD wounds were not.

### 3.2. Histology

**Figure 3:**
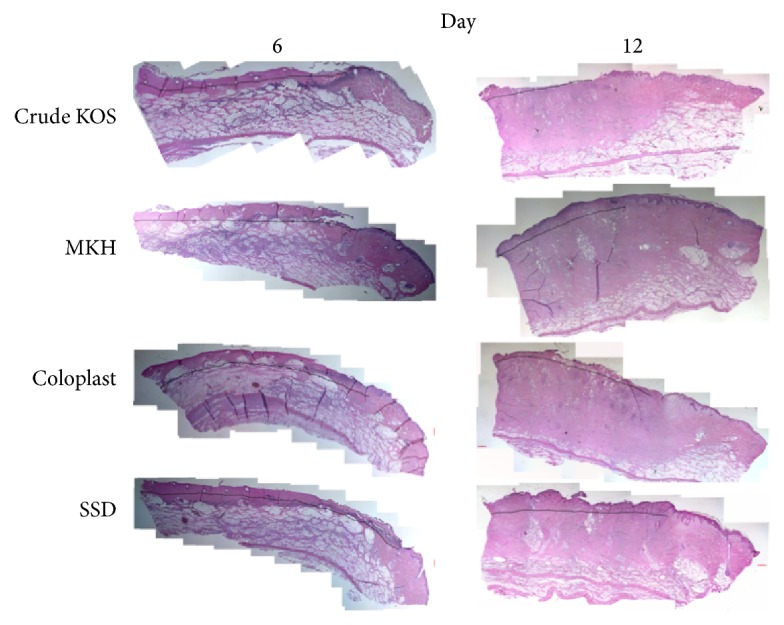
General histology. Images of H&E staining of representative tissue samples from each experimental group at days 6 and 12.

General histology images are shown in Figure 3.

#### 3.2.1. Burn Depth

**Figure 4:**
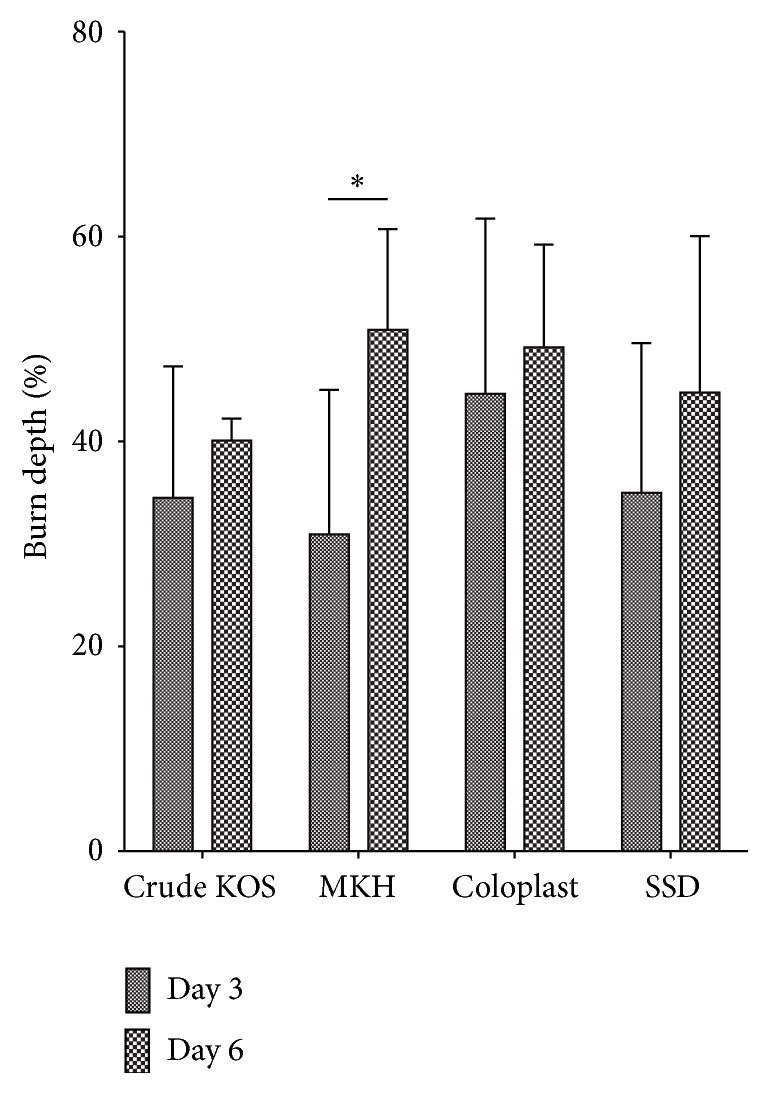
Burn depth. Vertical progression in burn depth was observed for all treatments between days 3 and 6, but only the MKH treatment group showed statistical significance. Crude KOS and Coloplast appeared to show the least burn depth progression (^*∗*^ *P* < 0.05; *n* = 6 across 2 pigs at each time point).

On day 3, the average burn depth was similar in all treatment groups. The Coloplast group was slightly deeper, but this difference was not statistically significant. By day 6, all wounds had increased in depth, but by a statistically significant amount only in the MKH treatment group (Figure 4). By day 9, vertical progression was evident in all the treatments. Crude KOS treatment (5.56%) had a minimal increase in burn depth compared to the other treatments (Coloplast: 22.5%, MKH: 20.01%, and SSD: 9.81%) (Figure 2 and Table 1). However, there was no statistically significant difference in burn depth between the treatments.

#### 3.2.2. Thickness of Granulation Tissue

**Figure 5:**
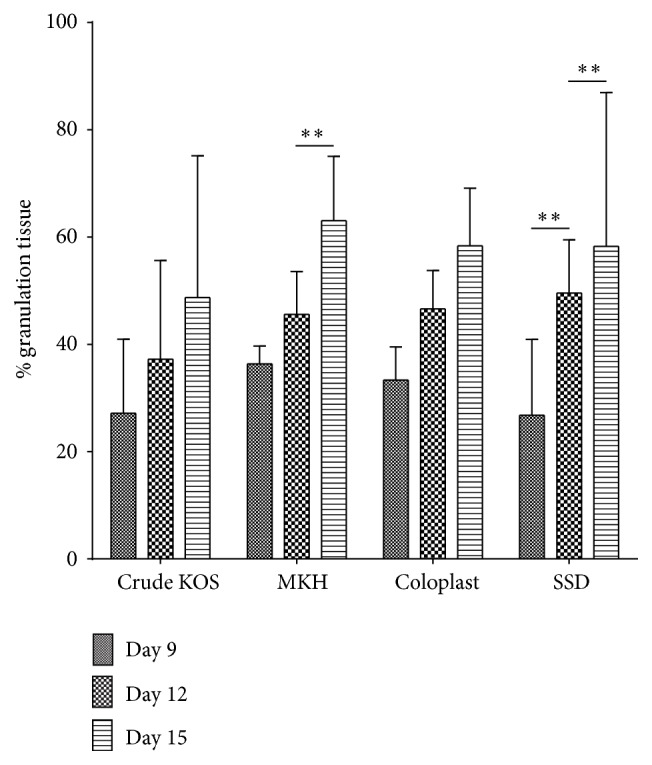
Granulation tissue assessment. SSD treatment resulted in significant increases in granulation tissue from days 9 to 12 and from 12 to 15. MKH treatment resulted in significant increases in granulation tissue from days 12 to 15 (^*∗∗*^ *P* < 0.01; *n* = 6 across 2 pigs at each time point).

SSD treatment showed the largest difference in granulation tissue, with significant increases from days 9 to 12 and from days 12 to 15 (*P* < 0.01) (Figure 5). While the percent granulation tissue increased in all other groups as well, these increases were not statistically significant.

#### 3.2.3. Reepithelialization

**Figure 6:**
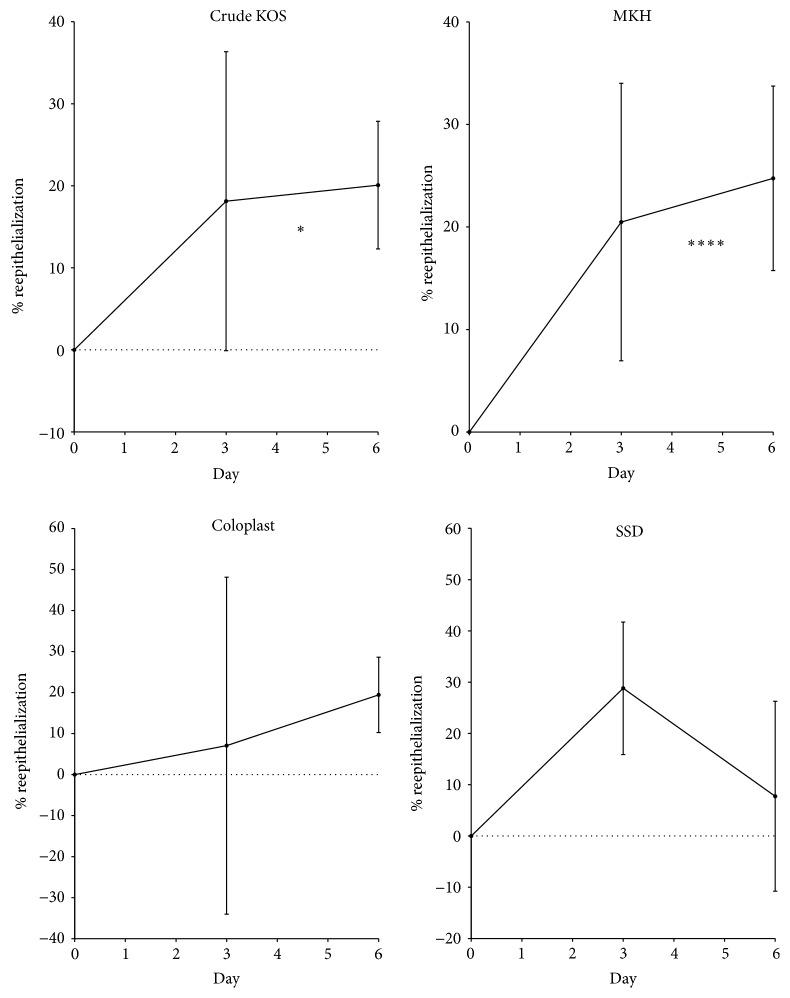
Early time points of reepithelialization. Burns treated with Crude KOS or MKH treatment demonstrated a general upward trend in reepithelialization at early stages of wound reepithelialization, whereas SSD generally trended downward and Coloplast showed large variation. There was a statistically significant difference from day 0 to day 6 (i.e., positive slope) in the Crude KOS and MKH treatments but not in the Coloplast and SSD treatments (^*∗*^ *P* < 0.05; ^*∗∗∗∗*^ *P* < 0.001; *n* = 6 across 2 pigs at each time point).

**Figure 7:**
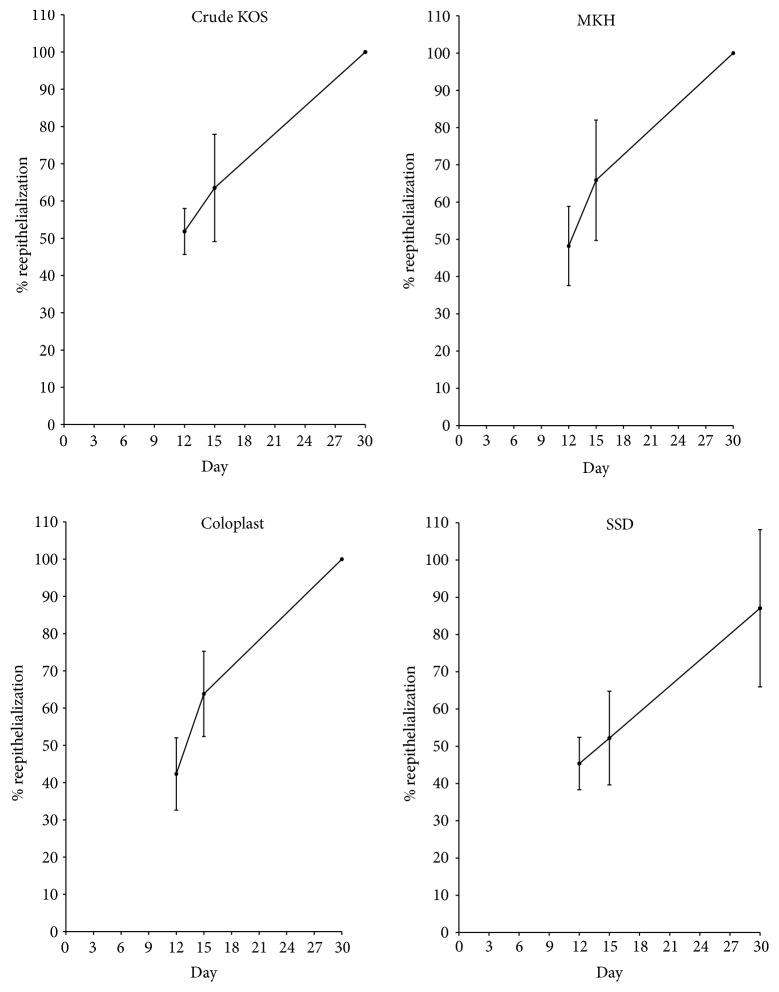
Later time points of reepithelialization. Compared to the SSD treatment, Crude KOS, MKH, and Coloplast treatments appear to promote faster reepithelialization at later time points. Burns treated with hydrogels reached complete wound closure by 30 days, while SSD treated wounds had not (*n* = 6 across 2 pigs at each time point).

Burns treated with Crude KOS and MKH demonstrated a statistically significant upward trend in reepithelialization at early time points (i.e., through day 6; *P* < 0.05 and 0.01, resp.) while Coloplast and SSD did not (Figure 6). For SSD, this observation is consistent with previous findings that SSD can impede reepithelialization [20]. At later time points (days 12, 15, and 30), the rate of reepithelialization was not statistically different between any of the treatment groups (Figure 7). Regardless, early differences in reepithelialization appeared to contribute to a differential overall wound closure rate wherein all biomaterial treatments required approximately 25 days to completely close while SSD treated wounds required almost 35 days (Table 1).

## 4. Discussion

Due to tissue necrosis and burn wound progression, deep partial thickness burns can convert to full thickness burns, thereby increasing risk to the patient, length of hospital stay, and the costs associated with treatment. At the early stages after burn injury, the processes of healing and wound progression are in opposition to each other, and most often progression prevails due to a lack of specific treatments that can address tissue loss at the level of cellular pathways. Currently, there are no approved treatments that can control burn injury progression by regulating death signals within the cell. The standard of care is generally focused on avoiding infection while allowing the burn to take its natural course, followed by excision of dead tissue after some short time period to facilitate healing from the healthy periphery of the wound. However, the standard of care, SSD, did not perform as well as the other treatments in this study. Despite its beneficial antimicrobial properties [21–23], the use of SSD as a standard of care has been questioned due to its reported interference with reepithelialization [20]. Tian et al. suggested the use of silver nanoparticles as a more effective alternative to SSD [24], and crystalline silver has also been shown to have beneficial wound healing properties [25], but to our knowledge no comparative testing of these different forms of silver in the same burn model has been published. Indeed, the present study showed the primary issue with the use of SSD to be delayed reepithelialization and, hence, a longer time to wound closure. By all other outcome measures, however, SSD appeared to perform similar to the hydrogel treatments.

Assessment of digital images of wound areas shows that both keratin hydrogel treatments, Crude KOS and MKH, produced a significant difference in wound size, but only at day 12. Coloplast, a collagen-based hydrogel, showed significant improvement compared to SSD at days 9 and 12 (Figure 1), but no differences compared to either keratin hydrogel treatment. Closer inspection of reepithelialization data at early time points (Figure 6) reveals statistically significant epithelial growth only in the keratin hydrogel treatments, but not for Coloplast (or SSD). These data suggest that the hydrogel treatments are having a beneficial effect, but perhaps for different reasons. Detailed assessment of the wound size data shows that burns increased in area from burning to day 3 in all groups, but less so for Coloplast. However, there was high variability in wound size in the Coloplast treatment group at this early stage with a standard deviation nearly three times that of Crude KOS, MKH, and SSD, thereby negating any statistically based conclusions. These observations cannot be explained by wound contraction as all burns were tattooed and tracked with no significant contraction occurring in any treatment group (data not shown). Later stages of reepithelialization appeared to be similar in all treatment groups (Figure 7), leading to a close clustering of days to wound closure data (Table 1). SSD is slightly longer than other treatments, but there is no statistical significance in these data.

As expected, the injury model did demonstrate burn injury progression in all treatment groups, but it was the Crude KOS that showed the smallest increase in burn depth compared to the other treatments, particularly in the vertical direction. This finding may indirectly support our hypothesis as the Crude KOS formulation contained the highest level of gamma keratose, which may be the reason that vertical progression was relatively lower (although not statistically significant). While we postulated that a lower concentration of gamma keratose in the gel would be sufficient, the release kinetics of these active peptides were not studied prior to the initiation of the present study and could have been lower than expected.

Granulation tissue depth, a measure of later stages of healing, was highest in the SSD group. However, this may have been due to the fact that this treatment group experienced the most vertical wound progression and consequently had the most extensive tissue loss. Higher amounts of granulation tissue may have been the normal response to tissue loss and not necessarily indicative of better healing. Regardless, the differences in this outcome measure were again found to lack statistical significance when comparing the different treatments, largely due to the variability in the SSD treatment group.

In order to better understand the apparent lack of statistical power in our experimental design, power calculations were performed based on the reepithelialization data. These showed that two more replicates would be required to show a significant (*P* < 0.05) difference in reepithelialization rate between MKH and SSD, and more than 100 replicates would be required to show a significant difference between MKH and Crude KOS or Coloplast. These numbers point toward improvement in arguably the most important outcome in burn treatment, reepithelialization, but they do not necessarily support the conclusion that the amount of gamma keratose was optimal and/or that the route of administration was ideal in this study. As was shown previously [14], the gamma keratose fraction contains large peptide molecules (i.e., fragments of alpha keratins) and delivery of these compounds through the skin and especially through a thick layer of eschar would be expected to be difficult and inefficient. Interestingly, Nunez et al. have shown that soluble keratose has vasodilation properties when administered intravenously or topically [26]. Considering that the zone of stasis is characterized by reduced blood circulation due to the clogging of blood vessels after burn injury [2], perhaps part of the mechanism of tissue sparing may be due to keratin's ability to dilate blood vessels in the tissue surrounding the burn. Obviously, a better understanding of the mechanism of action, optimal dose, and efficient administration of the most efficacious keratin compounds will require more research.

## 5. Conclusion

In this study, we aimed to understand if a particular formulation of keratin hydrogel containing a reduced concentration of gamma keratose, MKH, could have a beneficial effect on burn wound healing. We compared MKH to Crude KOS that was previously shown to promote burn wound healing and that contained a relatively large fraction (i.e., ca. 20%) of peptides, known as gamma keratose, that rescued thermally stressed cells. The amount of gamma keratose was reduced in MKH to reflect the concentration that demonstrated efficacy in a cell culture model of thermal stress. A statistically significant difference in wound size was observed, but this did not translate to the histological measurements. The rate of reepithelialization appeared faster in the MKH treatment group at early time points where it was expected to have its greatest effect, but statistical significance was lost due to high variance in the SSD treatment group. Regardless, the outcome measures in this study were not able to differentiate MKH (i.e., low gamma keratose concentration) from Crude KOS (high gamma keratose concentration). More research to investigate the mechanism of keratins in burn wound healing and more specifically in burn injury progression is needed.

## Figures and Tables

**Table 1:** Days to wound closure. Crude KOS, MKH, and Coloplast treatments appear to show faster wound closure compared to SSD.

Treatment	Mean days to wound closure
Coloplast	24.2 ± 7.3
Crude KOS	25.0 ± 4.9
MKH	25.8 ± 4.6
SSD	34.6 ± 7.6

## Abbreviations

KAP: Keratin associated proteins
cm: Centimeter
*μ*m: Micrometer
PEG: Polyethylene glycol
kg: Kilograms
mg: Milligrams
mcg/h: Micrograms per hour
DI: Deionized (water)
IPA: Isopropyl alcohol
H&E: Hematoxylin and eosin
*α*: Alpha
*γ*: Gamma
APC: Activated protein C
r-TPA: Recombinant tissue type plasminogen
rpm: Revolutions per minute
RCF: Relative centrifugal force
MRad: Mega radiation absorbed dose
kDa: Kilodalton
PBS: Phosphate buffer saline.
